# Immediate Postpartum Long-Acting Reversible Contraception: A Comparison Across Six Humanitarian Country Contexts

**DOI:** 10.3389/fgwh.2021.613338

**Published:** 2021-04-06

**Authors:** Meghan C. Gallagher, Catherine N. Morris, Aisha Fatima, Rebekah W. Daniel, Abdikani Hirsi Shire, Bibiche Malilo Matala Sangwa

**Affiliations:** ^1^Save the Children USA, Department of Global Health, Washington, DC, United States; ^2^Save the Children International, Pakistan Country Office, Islamabad, Pakistan; ^3^UNC Gillings School of Public Health, Chapel Hill, NC, United States; ^4^Save the Children International, Somalia Country Office, Puntland, Somalia; ^5^Save the Children International, Democratic Republic of Congo Country Office, Goma, Democratic Republic of Congo

**Keywords:** humanitarian settings, immediate postpartum contraception, intrauterime device, implant, acute crisis, protracted emergencies, Long acting reversible contraception (LARC), healthy timing and spacing of pregnancy

## Abstract

Postpartum family planning (FP) could prevent more than 30% of maternal deaths by effectively spacing births; this is particularly relevant in humanitarian contexts given that disproportionate maternal death occurs in countries affected by crises. In humanitarian settings, where accessing functional facilities is challenging with security risks that constrain movement, many women are unable to return for their 6-week postpartum visits and thus unable to receive FP counseling and adopt a method that suits their fertility intentions. Thus, immediate postpartum family planning (IPPFP) interventions, focused on long-acting reversible contraception (LARC) and tailored toward humanitarian contexts, could contribute to healthy timing and spacing of pregnancy, particularly among postpartum women, and improve maternal and newborn health. In 2014, Save the Children integrated postpartum intrauterine device (IUD) services into its FP package in emergency settings. In 2017, this expanded to include postpartum implant uptake as well, given updated World Health Organization guidelines. Three countries (Democratic Republic of Congo, Somalia, and Pakistan) opted for higher-intensity programming for IPPFP with a specific focus on LARC. This involved training delivery-room providers on counseling and provision of IPPFP, as well as training antenatal care nurses in counseling pregnant women on IPPFP options. Three countries (Rwanda, Syria, and Yemen) did not implement notable IPPFP interventions, although they provided the standard of care and monitored provision via monthly service delivery data. Using data from 2016 to 2019, we examined trends in immediate postpartum LARC (IPP LARC) uptake and compared countries with higher-intensity IPP LARC interventions to countries providing standard care. Tests of association were performed to assess the significance of these differences. In the country programs with higher-intensity IPPFP interventions, IPP LARC as a percentage of all deliveries was much higher overall during the July 2016–December 2019 period. The IPP LARC intervention had a significant impact on the overall proportion of women and girls who adopted an IUD or implant within the first 48 h of delivery, *F*_(1, 250)_ = 523.16, *p* < 0.001. The mean percentage of IPP LARC among all deliveries in intervention country programs was 10.01% as compared to 0.77% in countries providing standard care. Results suggest that there is demand for IPP LARC in humanitarian contexts and that uptake increases when multipronged solutions focusing on provider training, community outreach, and service integration are applied.

## Introduction

Postpartum family planning (PPFP) could prevent more than 30% of maternal deaths by effectively spacing births (1). Given that more than 60% of maternal deaths occur in countries affected by humanitarian crises, it is necessary to understand the drivers for and barriers to increasing demand and use of PPFP in countries affected by conflict and natural disaster (2).

Humanitarian settings, also referred to as conflict-affected or emergency settings, can be a direct result of human-induced conflicts, such as war and economic insecurity, or environmental conflicts, such as cyclones, droughts, and famine. These crises can also be further described as a *protracted crisis*, which is characterized by recurrent or long-lasting conflict or persistent emergency, or *acute crisis*, which is characterized as an active conflict or natural disaster. Displacement, exposure to environmental toxins, persecution, and gender-based violence are just some of the many devastating conditions experienced by communities in these settings. In some cases, protection of marginalized groups by a national government is not guaranteed, often culminating in human rights violations.

As of 2017, at least 129 million people around the globe live in humanitarian settings, with nearly one-fourth being women and adolescent girls of reproductive age (3). The structural instability and limited resources in humanitarian settings can make it particularly difficult to obtain adequate FP services, much less PPFP services. A mere 16% of sexual and reproductive health (SRH) services in conflict-affected settings in sub-Saharan Africa are able to provide comprehensive FP services (4). And although more than 95% of postpartum women in resource-limited countries did not want another pregnancy within a year, nearly half had an unmet need for FP services (5). These gaps in services can be problematic as it suggests high rates of unplanned pregnancies, complicated births, unsafe abortions, and even higher mortality rates in humanitarian settings (3, 6).

Family Planning High Impact Practices, guidance used for FP delivery primarily in development settings, recommends offering contraceptive counseling and immediate postpartum family planning (IPPFP) services as part of facility-based childbirth care prior to discharge from the health facility (7). In humanitarian settings, where accessing functional facilities is challenging with security risks that constrain movement, many women are unable to return for their 6-week postpartum visits and thus unable to receive FP counseling and adopt a method that suits their fertility intentions. Thus, IPPFP interventions tailored toward humanitarian contexts could contribute to improvements in FP uptake, particularly among postpartum women, and reduce maternal mortality in crisis settings (8, 9).

Family planning, inclusive of the postpartum period, allows women to exercise their human right to contraceptive access so they may choose when to conceive as well as prevent unintended pregnancies if they choose not to conceive (10). By practicing this rights-based approach, women can ensure the healthy timing and spacing of pregnancy (HTSP), which is a notable benefit of PPFP (11). HTSP reduces the risks of adverse maternal and perinatal outcomes by giving the mother enough time to recuperate after her delivery and provide care for her child. Contrarily, short intervals between pregnancies increase the likelihood of maternal mortality, subsequent premature and low-birth-weight babies, congenital disorders, and other birth-related complications (12–14). Nonetheless, the unmet need for contraception among postpartum women remains high in many resource-limited settings (15).

Integrating high-impact practices such as immediate postpartum IPPFP counselling services and services into FP programs is key in addressing gaps in unmet FP needs (7). The World Health Organization (WHO) Medical Eligibility Criteria for Contraceptive Use (MEC) provides guidance on the safety of contraceptive methods for use given specific characteristics and health contexts using numerical categories ranging from one (a condition for which there is no restriction for the use of the contraceptive method) to four (a condition that represents an unacceptable health risk if the contraceptive method is used) (16). Contraceptive methods that can be performed or used immediately after childbirth (i.e., within 48 h of delivery) and are appropriate for women who are breastfeeding have MEC categories of 1 and 2. These include the intrauterine device (IUD) within 48 h of delivery (copper-bearing is MEC category 1, levonorgestrel is MEC category 2); implants (MEC category 2), progestin-only pills (MEC category 2), lactational amenorrhea method (LAM), condoms, and voluntary surgical contraception (VSC) (16).

Progestin-only pills, condoms, and LAM are highly effective methods when used adherently and consistently. VSC is a permanent method that requires highly skilled healthcare personnel and explicit service provision when implemented into practice (11), given the uncertainty that often exists within humanitarian contexts that challenges contraceptive adherence along with the common shortage of health providers with the skills and qualifications needed to perform VSC. Both IUDs and contraceptive implants are long-acting reversible contraceptive (LARC) methods that are effective for anywhere between 3 and 10 years, depending on the type of LARC inserted. The IPP period has several potential benefits for LARC use as women know they are not pregnant and are motivated to avoid short-interval pregnancy. In the case of an IUD, given that the uterus is often lax during the IPP period, insertion can be much easier, and less painful after childbirth (17). The discomfort related to standard insertion can also be masked by the lochia, vaginal bleeding, and discharge, which occurs during and shortly after childbirth (17). An additional benefit of IPP LARC use is that clients are already under the care of a medical provider when giving birth, so it proves to be more cost-effective and provides a level of convenience to the client when inserted after childbirth (18, 19). The efficacy of LARC methods combined with the immediacy of insertion allows for HTSP and ultimately improves maternal, newborn, and child health (15). With the assumption of free and informed choice, LARCs have been shown to be a safe, effective, and feasible IPPFP intervention for HTSP (20).

Immediate LARC insertion can be beneficial to clients who face access barriers or are unable to follow up with postpartum care visits as recommended, which can be especially relevant in humanitarian settings because of the structural instability and a woman's desire to delay pregnancy because of conditions of conflict and uncertainty (12, 14, 21). With that said, IPPFP can negligently be viewed as low priority when providing humanitarian aid in these contexts despite a continued need and desire for HTSP (22, 23).

While the topic of IPPFP has become an emerging interest among FP initiatives and humanitarian workers, there remain gaps in understanding its application in conflict settings. The benefits of IPPFP are contingent on its effective implementation, which requires quality clinical training, availability of supplies and commodities, community sensitization, and the installation of monitoring systems.

**Figure 1:**
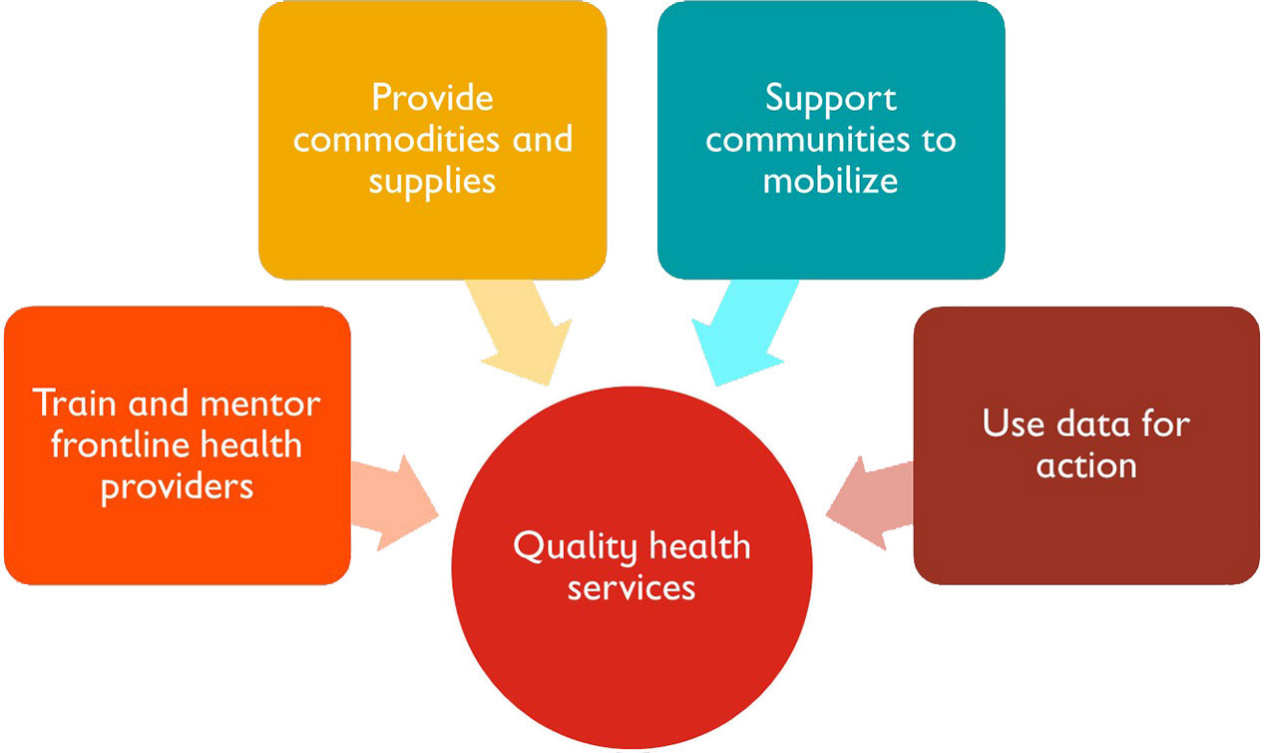
Save the Children's approach to ensure voluntary, high-quality FP, and PAC services in humanitarian settings.

To address the issue of high maternal morbidity and mortality among women and girls living in humanitarian settings, Save the Children began implementing a SRH program focused on FP and postabortion care (PAC) in 2012. Working in collaboration with the Ministries of Health, governments, and other relevant stakeholders, Save the Children applied a four-pronged approach to ensure voluntary, high-quality FP and PAC services in humanitarian settings; this included capacity building, assurance of supplies and infrastructure, community collaboration and mobilization, and consistent data management for ongoing monitoring, evaluation, and data use (Figure 1).

To better support HTSP and improve access to PPFP, Save the Children integrated voluntary IPP IUD services into its FP package in emergency settings in 2014. Given Save the Children's extensive work in maternal and newborn health, IPP contraception provided yet another opportunity for women, girls, and families to learn about contraception options that suited their fertility desires. In 2016, program indicators for tracking IPP IUD use were standardized and systematically monitored across country portfolios. In 2017, as a result of updated guidance from the WHO, Save the Children expanded the package to include implant insertion within the first 48 h of delivery. This gave women and girls greater choice and provided the opportunity to expand IPPFP services.

Within the broader package of offered SRH services, country programs prioritized activities based on population needs, community norms, and health system capacity. Three countries (Democratic Republic of Congo, Somalia, and Pakistan) opted for higher-intensity programming for voluntary IPPFP. Three countries (Rwanda, Syria, and Yemen) did not focus as intensely on IPPFP-specific activities and interventions; however, IPPFP was a component of standard care within the overall FP program; thus, providers were trained on IPPFP, relevant supplies were available, and IPPFP service delivery data were monitored on a monthly basis.

We hypothesized that the countries focused more intensively on expanding and improving IPPFP services would see heighted demand for IUD and implant insertion within the first 48 h of delivery. Taking into account varied IPPFP programmatic approaches, we compare country programs with higher-intensity IPPFP interventions to countries who maintained standard care.

## Materials and Methods

To evaluate the overall uptake of IPP LARC within six country programs, we analyzed the retrospective service delivery data from each of the country programs from July 2016 through December 2019, examining overall trends and comparing programs with higher-intensity IPPFP interventions to those applying standard care.

The six country programs are diverse in their geography, populations served, contraceptive prevalence, and contraceptive method mix (Table 1) (24). Table 1 notes when each of the six Save the Children FP programs commenced, the number of facilities supported, and the overall program catchment areas. Using data from The United Nations Department of Economic and Social Affairs' *Contraceptive Use by Method 2019*, the table also notes contraceptive prevalence and overall method mix for each of the six countries at a national level. In many instances, the national method mix included in Table 1 is not reflective of the method mix within the country program geographies; further, many national surveys are out of date and do not fully capture the areas within countries that are affected by crises. The Democratic Republic of Congo (DRC) data derived from 29 health facilities in rural Masisi and Mweso in North Kivu; Pakistan from 8 urban and periurban health facilities in Shikarpur and Jacobabad; Somalia from 10 rural health facilities in Puntland; Rwanda from one rural health facility within a Burundian refugee camp in Kirehe; Syria from 6 urban health facilities in Aleppo and Idlib; and Yemen from 16 urban health facilities in Lahj and Hodeida.

**Table 1:** Overview of family planning programs in six countries.

**Save the Children program information**				**National-level contraceptive use data Contraceptive use by method 2019; United Nations Department of Economic and Social Affairs**									
	**Program commencement**	**No. health facilities**	**Catchment population**	**Any method^1^ (%)**	**IUD (%)**	**Implant (%)**	**Injectable (%)**	**Pill (%)**	**F. sterilization (%)**	**M. Sterilization (%)**	**Male condom (%)**	**Other^2^ (%)**	**Year of latest survey data**
DRC	2012	29	493,948	22.4	0.2	0.8	1.3	1.0	0.7	0.1	6.8	11.6	2013
Pakistan	2012	8	799,773	23.6	1.5	0.3	1.8	1.2	6.4	0.1	6.4	5.9	2017
Somalia	2012	10	385,851	14.9	0.5	0.1	1.1	3.7	0.0	0.0	0.2	9.3	2006
Rwanda (Burundian Refugees)	2016	1	28,762	19.4^*^	0.7^*^	4.6^*^	8.6^*^	1.2^*^	0.4^*^	0.1^*^	1.3^*^	2.7^*^	2016^*^
Syria	2016	6	315,000	31.6	14.1	0.0	0.6	5.5	1.9	0.0	1.6	7.9	2009
Yemen	2013	16	507,489	25.5	4.3	0.4	3.0	8.4	1.9	0.1	0.6	14.6	2013

1 *Source: United Nations, Department of Economic and Social Affairs, Population Division (2019). Contraceptive Use by Method 2019: Data Booklet (ST/ESA/SER.A/435)*.

2 *Other category includes rhythm method, withdrawal, lactational amenorrhea method, and various other cited methods*.

* *Burundi country data*.

All six country programs received standard and comparable support for FP service delivery in terms of clinical capacity building, assurance of supplies and appropriate infrastructure, demand creation, and support on data collection and management. The higher-intensity IPPFP intervention applied in DRC, Pakistan, and Somalia focused on three overall facets of voluntary IPPFP service delivery- clinical training, health facility operations/service integration, and community mobilization. Although IPPFP was a component of clinical training for all country programs, a rights-based cascade training specifically for IPPFP was effectively rolled out in the DRC, Pakistan, and Somalia. After a 5-day training-of-trainers, master trainers trained health providers locally, with a focus on midwives and nurses within maternity departments at supported facilities. Task sharing was essential, given that facilities are often understaffed and do not always have medical doctors consistently on-site. On-the-job training and mentoring proved very useful in ensuring IPPFP quality and motivating providers. There was a strong focus on IPPFP-specific counseling, during antenatal care (ANC) visits and within maternity departments where women and girls are waiting prior to labor and delivery. It was critical that IPPFP counseling provided accurate information that allowed women and girls the right to make a free and informed choice. IPPFP-specific IEC materials were created and were available for outpatient visits, ANC visits, FP visits, and within maternity departments.

Operational changes were made at facilities to better support IPPFP services. Programs ensured that counseling materials and implant and IUD kits, for both insertion and removal, were available within maternity departments, especially in health facilities where FP services are often separated from maternal health services. Further, IPPFP counseling during ANC required linkages to labor and delivery as well. In Pakistan, a tracking system was developed so that those who expressed interest in IPPFP during ANC visits would automatically be flagged for further IPPFP counseling and preparation within the maternity department. In order to increase demand for IPPFP, quality is critical. The country programs closely monitored IPPFP services and reasons for LARC removal to ensure that there were no issues with infection or perforation.

Finally, the DRC, Somalia, and Pakistan country programs conducted community outreach to create further understanding of and demand for IPPFP services so that women, girls, and families could voluntarily choose a method of IPPFP if they desired. Community health workers incorporated IPPFP information into their ongoing health conversations. Male spouses and partners were encouraged to join for ANC visits so that they could better understand the benefits of IPPFP for mothers, newborns, and families. Within communities, IPPFP champions, both male and female, were identified to speak about their positive experiences with IPPFP and were available to meet with their neighbors to address any questions or concerns. IPPFP data were monitored on a monthly basis, and program modifications were made based on facility trends.

Retrospective FP data collected on a monthly basis were extracted for programs in the DRC, Pakistan, Somalia, Rwanda, Syria, and Yemen. To ensure data quality in all six country programs, Save the Children trained health providers, key Ministry of Health staff, and relevant Save the Children health staff on effective data management and data use. To reduce error, data were entered into a deployable DHIS-2 database and reviewed on a monthly basis to note unusual trends or discrepancies. Extracted variables of interest included the number of clients who adopted an IUD within 48 h of delivery (inclusive of postcesarean IUD, IUD insertion within 10 min of normal delivery, and IUD after 10 min and within 48 h of normal delivery), number of clients who adopted an implant within the first 48 h of delivery, total number of deliveries, number of new IUD users, number of new implant users, and total number of new FP users (overall and by method). Key indicators included the proportion of IPP LARC among all deliveries, the proportion of IPP LARC among all new FP clients, proportion of IPP IUD users among all new IPP IUD users and total FP clients, and proportion of IPP implant users among all new implant users and total FP clients. All country programs had standardized age disaggregated data by 2018; thus, all data and indicators were available for the following four age categories in 2018–2019: 10–14, 15–19, 20–24, and 25 years and older.

Using Stata 15, tests of association were performed to assess differences between higher-IPPFP-intervention countries and standard-care countries for key indicators. An analysis of variance was used to compare the proportion of IPP LARC uptake between higher-intensity intervention programs and standard care programs, and a *post-hoc* Tukey test was used to compare each of the six country programs. Changes in service delivery trends over time were observed for key indicators. IPP IUD and IPP implant use were analyzed in the context of overall FP use and method mix within each country program. Finally, we analyzed the proportion of IPP IUD and IPP implant uptake among all deliveries by age group.

The study was approved by the Save the Children Ethical Review Committee and does not constitute human subjects research, given its reliance on service delivery statistics.

## Results

The patterns of IPP LARC use varied by age group across the six country programs (Table 2). As expected, women older than 24 years had the highest proportion of IPP LARC uptake within each country. In Pakistan, IPP LARC was almost exclusively used by women 25 years or older (96.43%); however, in DRC, Rwanda, and Somalia, there was a more notable age distribution among IPP LARC users, including among adolescents aged 15–19 years. Table 3 shows the percentage of IPP LARC uptake among all facility-based deliveries by country program and age group. Similar to overall proportions of IPP LARC use, the country programs in DRC, Rwanda, and Somalia saw higher percentages of adolescents who delivered babies adopting IPP LARC (7.55% in DRC; 9.94% in Somalia; and 1.20% in Rwanda) as compared to Pakistan, Syria, and Yemen.

**Table 2:** Age distribution of immediate postpartum LARC clients, by country program.

	**10–14**		**15–19**		**20–24**		**≥25**	
	**%**	***n***	**%**	***n***	**%**	***n***	**%**	***n***
DRC	0.03%	1	7.22%	235	20.14%	655	72.61%	2,362
Pakistan	0.00%	0	0.00%	0	3.57%	39	96.43%	1,053
Somalia	0.00%	0	9.73%	167	27.21%	467	63.05%	1,082
Rwanda	0.00%	0	10.53%	2	26.32%	5	63.16%	12
Syria	0.00%	0	1.59%	8	10.71%	54	87.70%	442
Yemen	0.00%	0	0.00%	0	34.48%	10	65.52%	19

**Table 3:** Percentage of immediate postpartum LARC uptake among all facility-based deliveries, by country program and age group.

	**10–14**	**15–19**	**20–24**	**≥25**
DRC	1.89%	5.66%	8.14%	15.86%
Pakistan	0.00%	0.00%	1.20%	8.01%
Somalia	0.00%	9.94%	12.44%	13.08%
Rwanda	0.00%	1.20%	0.87%	0.81%
Syria	0.00%	0.21%	0.99%	5.28%
Yemen	0.00%	0.00%	0.15%	0.20%

In the country programs with higher-intensity IPPFP interventions, IPP LARC as a percentage of all deliveries was much higher overall during the July 2016–December 2019 period. The IPPFP intervention had a significant impact on the overall proportion of women and girls who adopted an IUD or implant within the first 48 h of delivery, *F*_(1, 250)_ = 523.16, *p* < 0.001. The mean percentage of IPP LARC among all deliveries in higher-intensity intervention country programs was 10.01% as compared to 0.77% in countries with standard care. Overall, in the DRC, 10.68% of all women and girls delivering at supported facilities adopted IPP LARC; in Pakistan, this was 6.65%, and in Somalia, 12.71%. In the standard-care countries, these statistics were much lower, with 0.52% of all women and girls delivering in supported facilities in Rwanda adopting IPP LARC, 1.73% in Syria, and 0.07% in Yemen.

The proportion of IPP LARC uptake among all deliveries by country yielded significant variation among country programs, *F*_(5, 246)_ = 179.47, *p* < 0.001. Each of the intervention countries (DRC, Pakistan, and Somalia) differed significantly from all other countries (*p* < 0.01). Syria and Yemen differed (*p* = 0.05); however, Rwanda was not significantly different from Syria nor Yemen.

**Figure 2:**
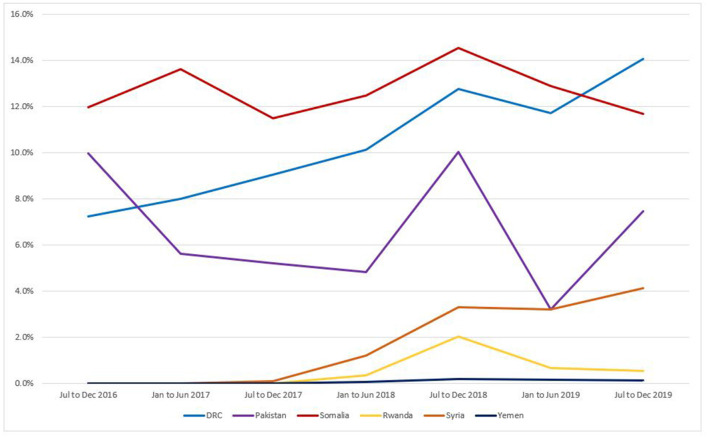
Immediate postpartum LARC update as a percentage of facility-based deliveries, July 2016-December 2019.

**Figure 3:**
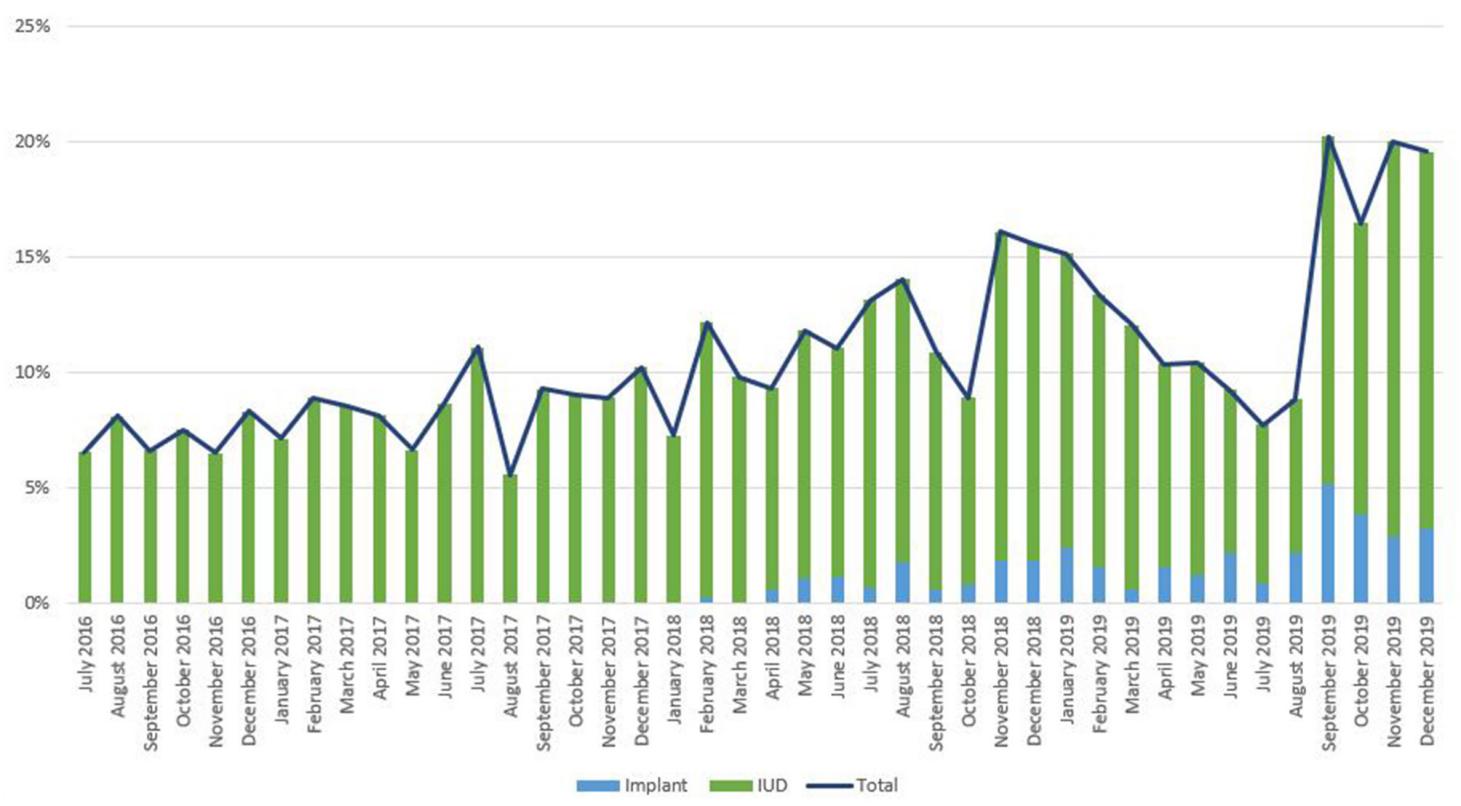
Immediate postpartum IUD and implant uptake among all facility-based deliveries, July 2016-December 2019, DRC.

**Figure 4:**
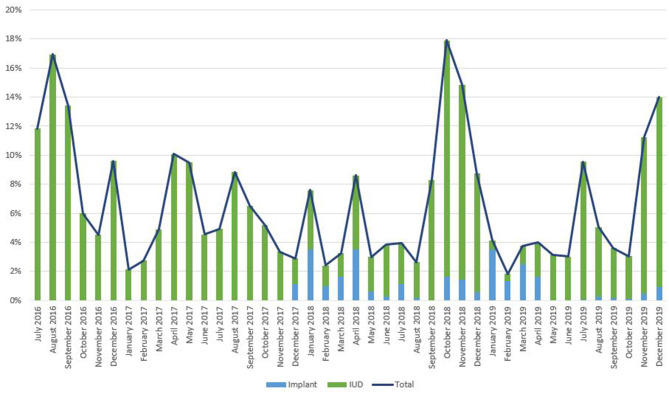
Immediate postpartum IUD and implant uptake among all facility-based deliveries, July 2016-December 2019, Pakistan.

**Figure 5:**
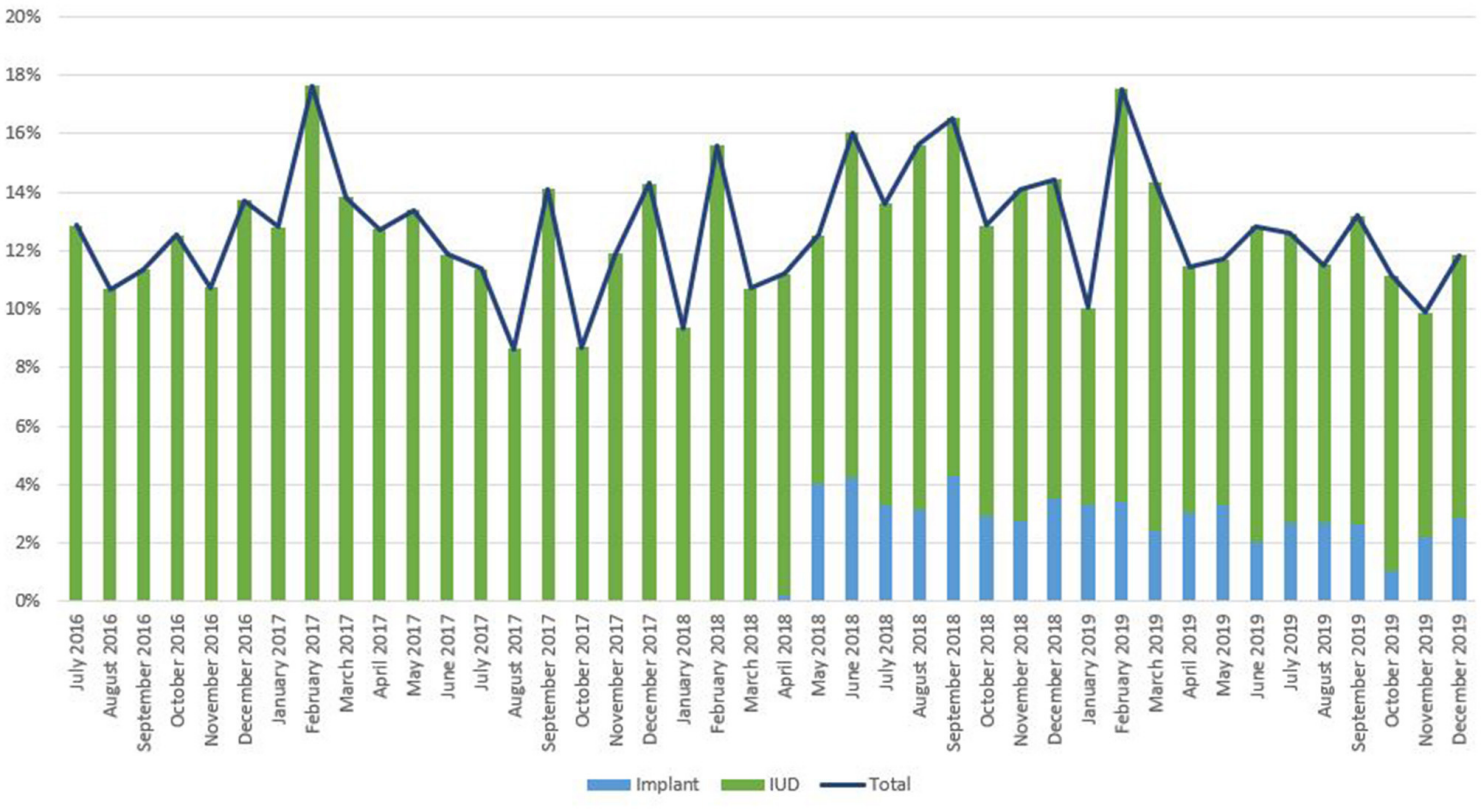
Immediate postpartum IUD and implant uptake among all facility-based deliveries, July 2016-December 2019, Somalia.

**Figure 6:**
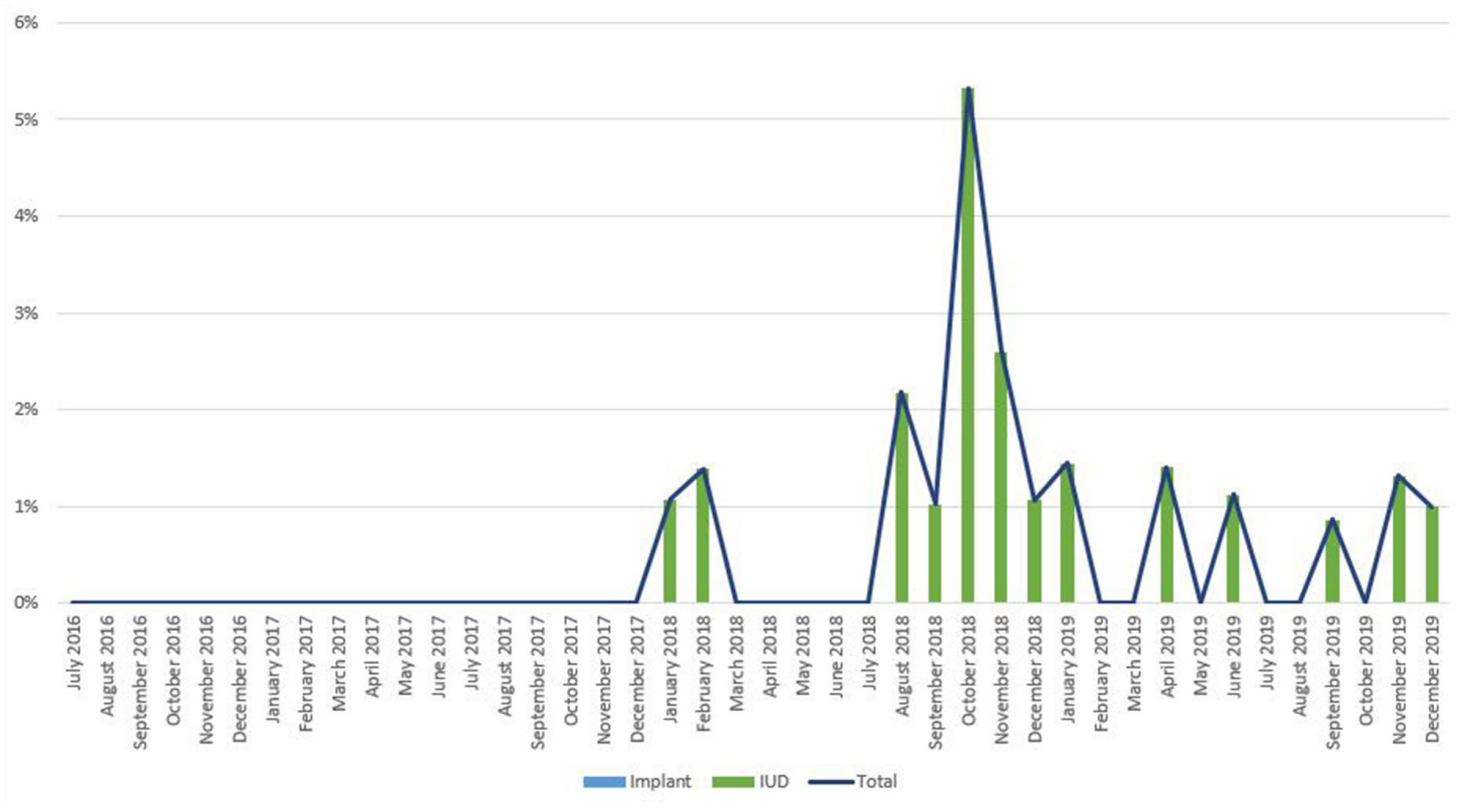
Immediate postpartum IUD and implant uptake among all facility-based deliveries, July 2016-December 2019, Rwanda.

**Figure 7:**
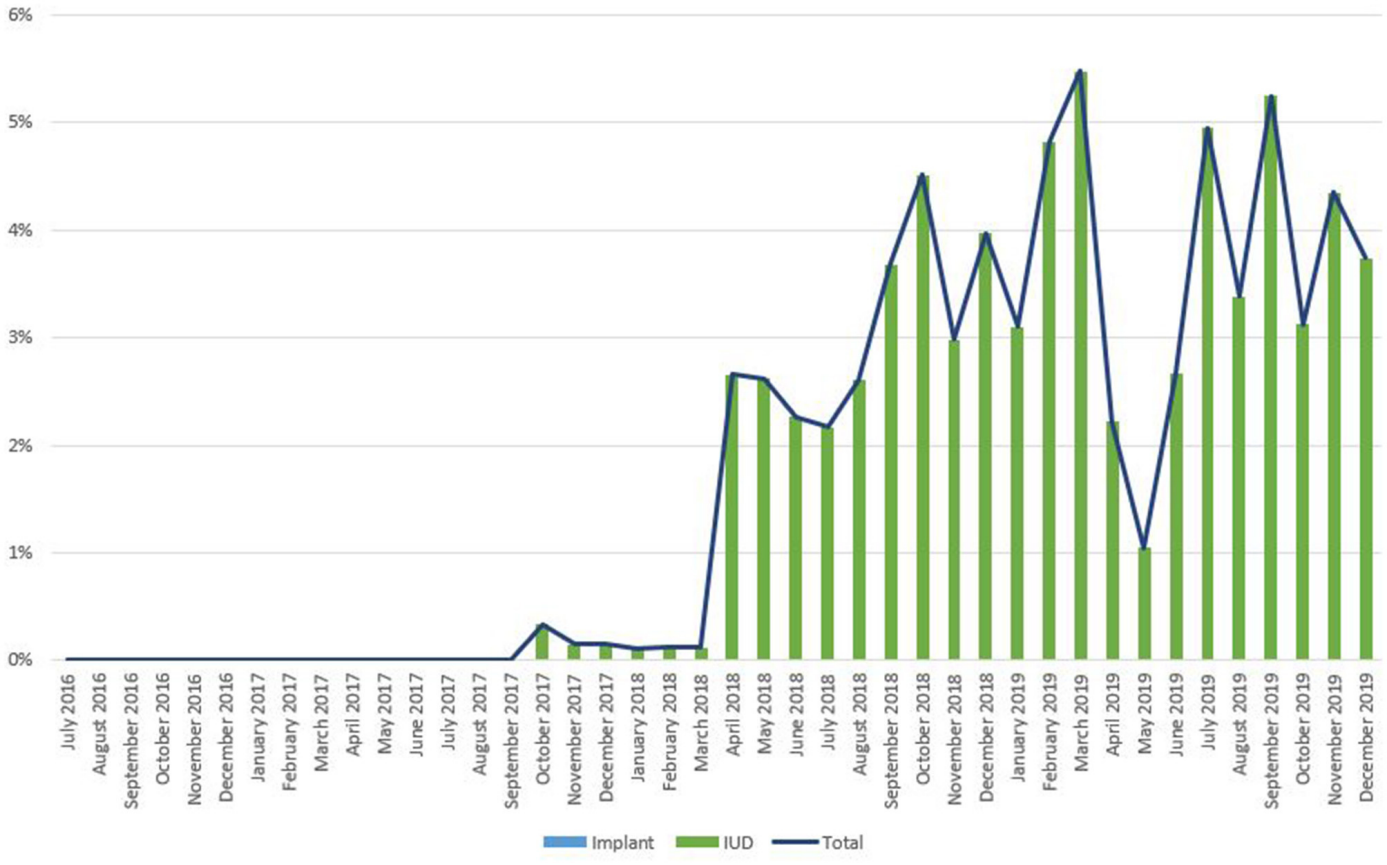
Immediate postpartum IUD and implant uptake among all facility-based deliveries, July 2016-December 2019, Syria.

**Figure 8:**
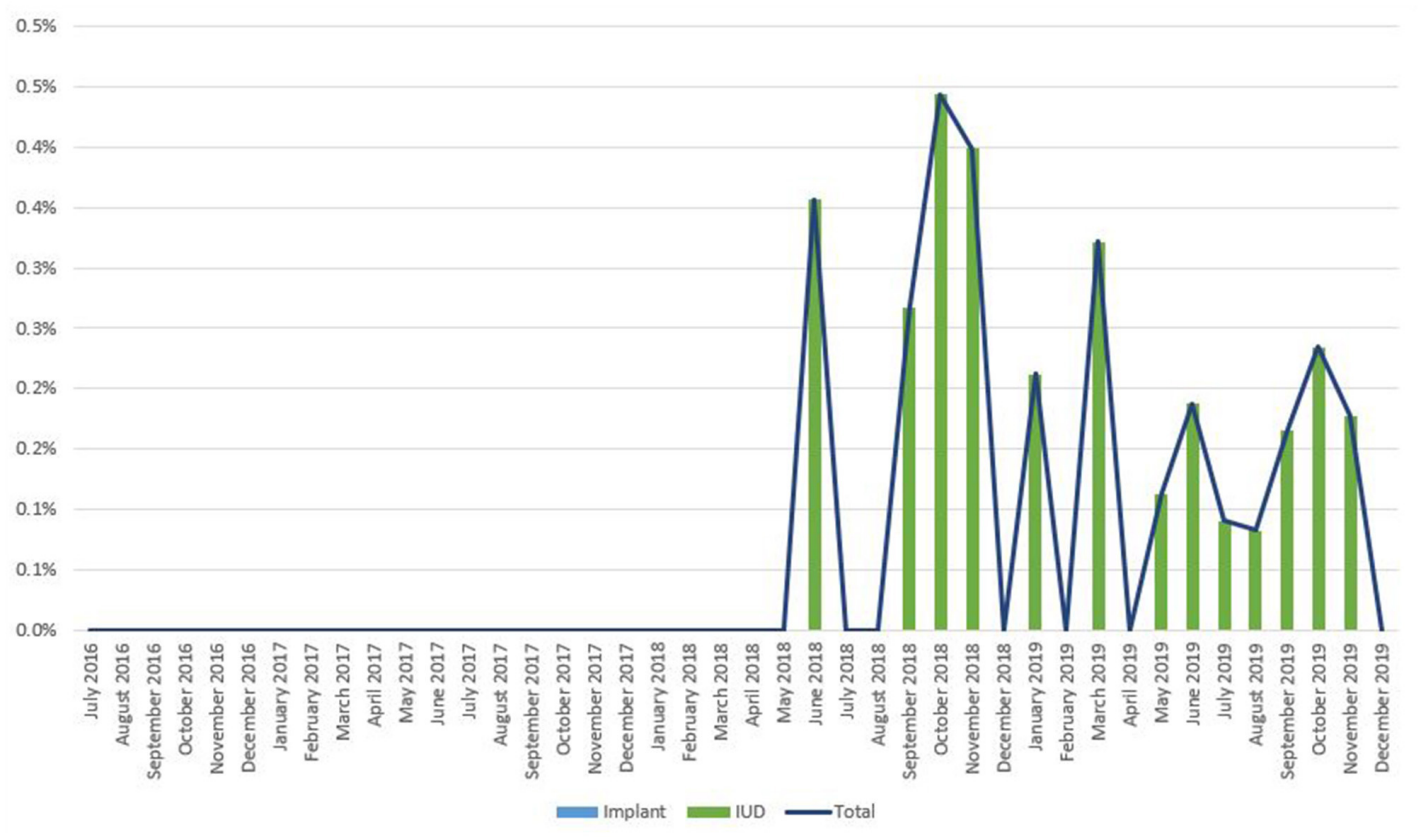
Immediate postpartum IUD and implant uptake among all facility-based deliveries, July 2016-December 2019, Yemen.

As noted in Figure 2, trends in IPP LARC contraception uptake varied across countries in time pattern and method. Figures 3–8 highlight monthly trends over time in percentage of IPPFP among all deliveries, as well as the proportion of IUD vs. implant IPPFP within each country program. In DRC, the percentage of IPPFP has trended upward over time; in July 2016, 6.5% of all women and girls delivering within a program-supported facility adopted an IPP IUD; and by December 2019, 19.6% of all clients delivering accepted IPP LARC. Ongoing training, community mobilization efforts, and the addition of implants into the IPP package has aided this trajectory in the DRC. Pakistan has seen spikes and dips in their IPPFP uptake overall due to quality training and focus on IPPFP accelerating services combined with consistent structural challenges due to FP services that were managed separately from maternal and newborn health services at the facilities. Pakistan saw the highest percentage of IPP LARC update in August 2016 (17.0%) and again in October 2018 (17.9%). Somalia, despite implants holding a much greater portion of the overall method mix, had early and sustained success with IPP IUD services; although implants now contribute to IPPFP services, overall IPPFP uptake remains fairly constant due to ongoing local training of midwives and systematic integration of IPPFP education and messaging into ANC visits. IPP LARC uptake in the Somalia program has been notably consistent, fluctuating between 8.7 and 17.6% of all deliveries. In Rwanda, Syria, and Yemen, there was an initial lag before IPPFP services were available. Although IPP IUD insertion had been a part of clinical training, specialized trainings on IPPFP were delayed for health providers in Yemen and Syria due to travel restrictions; these countries also experienced implant stock-out periods, making it difficult to fully incorporate implants into the IPPFP package. Syria began to see a steady increase in IPP IUD use in mid-2018, which continued to grow through the end of 2019, fluctuating between 2.6 and 5.5% of all deliveries. Rwanda's IPPFP uptake was inconsistent, although more prone to volatility given the small catchment area; when IPP LARC services were provided, uptake fluctuated between 1.3 and 2.2% of monthly deliveries. Yemen saw a very minor and uptick in July 2018 (0.36% of all deliveries), which was sustained through 2019, reaching a high of 0.44% of all deliveries in October 2018. Although availability of IPPFP has improved in Yemen, demand for services has consistently remained low.

In all countries, IUDs were a higher proportion of IPP LARC uptake as compared to implants (Table 4). However, with the exception of Syria and Yemen, implants comprise a greater proportion of the overall method mix. Both Syria and Yemen experienced implant supply delays during the July 2016–December 2018 period. DRC shows the most notable contrast, with nearly all IPPFP users opting for IUDs (93.1%, *n* = 4,817), despite IUDs comprising 15.1% (*n* = 9,615) of the overall method mix; meanwhile, implants comprise 53.1% (*n* = 33,779) of the overall method mix.

**Table 4:** Percentage of IUD and implant as proportion of IPPFP and overall method mix.

**Country**	**IUD percentage of overall IPPFP**	**IUD percentage in overall method mix**	**Implant percentage of overall IPPFP**	**Implant percentage in overall method mix**
DRC	93.1% (*n =* 4,817)	15.1% (*n =* 9,615)	6.9% (*n =* 358)	53.1% (*n =* 33,779)
Pakistan	89.5% (*n =* 1,337)	15.6% (*n =* 7,361)	10.5% (*n =* 157)	17.4% (*n =* 8,204)
Somalia	87.3% (*n =* 2,351)	14.6% (*n =* 4,785)	12.7% (*n =* 341)	15.6% (*n =* 5,103)
Rwanda	100% (*n =* 19)	4.5% (*n =* 216)	0.0% (*n =* 0)	24.6% (*n =* 1,175)
Syria	100% (*n =* 508)	34.1% (*n =* 7,549)	0.0% (*n =* 0)	1.7% (*n =* 372)
Yemen	100% (*n =* 32)	16.5% (*n =* 8,705)	0.0% (*n =* 0)	11.3% (*n =* 5,926)

Table 5 examines the contribution of IPP IUDs and IPP implants to implant, IUD, LARC, and FP use overall. It is notable that IPP IUD use contributes to approximately half of total IUD use in the DRC (50.10%) and Somalia (49.13%).

**Table 5:** Immediate postpartum IUD and implant contribution to overall family planning uptake, July 2016–December 2019.

**Country**	**IPP IUD as percentage of total new IUD acceptors**	**IPP implant as percentage of total new implant acceptors**	**IPPFP as percentage of total new LARC acceptors**	**IPPFP as percentage of total new FP acceptors overall**
DRC	50.10%	1.06%	11.93%	8.14%
Pakistan	18.16%	1.91%	9.60%	3.16%
Somalia	49.13%	6.68%	27.22%	8.24%
Rwanda	8.80%	0.00%	1.37%	0.40%
Syria	6.73%	0.00%	6.41%	2.29%
Yemen	0.37%	0.00%	0.22%	0.06%

## Discussion

This study shows that there is demand for IPP LARC in humanitarian contexts and that uptake increases when multipronged solutions focusing on provider training, community outreach, and service integration are applied. Proportion of IPP IUD vs IPP implant within each country program, increases in IPPFP uptake were seen within the July 2016–December 2019 period. In countries where IPPFP was prioritized within the broader package of contraceptive services, a greater proportion of women and girls opted for an IUD or implant within the first 48 h of their delivery. IPPFP was a larger proportion of overall LARC use in these higher-intensity countries. In places where short-acting methods are large proportions of the overall method mix, introducing IPPFP may be a way to expand uptake of LARC methods.

Even within country programs that successfully expanded services and demand for IPPFP, there were still challenges associated with implementation and sustainability. Sustainable human resources for health are a challenge worldwide, but notably in humanitarian settings (25, 26). Highly skilled health providers are often scarce and, as a result of war and natural disaster, cannot always remain at their assigned health facility. Although all facilities within each of the six program countries had providers trained on FP, these skills were often lost when providers left their post or took promotions elsewhere. To that end, applying a training-of-trainers model for IPPFP allowed for greater continuity and retention of these skills within health facilities, even during periods when provider availability was strained.

Integration of services was one of the more difficult challenges to overcome. For example, in Pakistan, FP services are provided under the direction of the Population Welfare Department, whereas the Department of Health focuses on maternal health, including emergency obstetric care and other reproductive health services. Because of the different management structures and operational policies, i.e., the Department of Health provides 24/7 care, it was important to involve both government entities in order to effectively ensure quality IPPFP services. Funding for IPPFP also proved challenging at times, given that it effectively needs to be incorporated into maternal and newborn health packages, but is often relegated to FP funding. In 2019, the Pakistan country program shifted its model to provide broader support to government health services, while scaling back the frequency of supervision; this type of shifting in funding and priorities can also lead to initial declines in service provision as new ways of working are established. Continued efforts to fund and support integrated health services across outpatient FP and inpatient labor departments will improve the delivery of IPPFP services, among other related maternal, newborn, and reproductive health services.

Access to IPPFP expanded the LARC portion of the overall method mix in places where standalone LARC uptake is a smaller portion of the overall method mix, notably Somalia and Pakistan. Similarly, IPPFP services prompted demand for IUDs, which remained the preferred IPPFP method over implants, even in places like the DRC and Somalia, where implants are a higher proportion of the method mix.

There are several limitations to this study, most notably its sole reliance on monthly service delivery statistics at a facility-level and program documentation. The client perspective is missing from this study, which limits our understanding of drivers for IPPFP services, notably in the way of LARC use within the first 48 h of delivery. Future qualitative studies among a diversity of clients would deepen evidence and understanding of IPPFP, notably among marginalized groups, including adolescents, people living with disabilities, and populations on the move. Further, there is an inherent selection bias in the study given that the country programs with higher-intensity IPPFP interventions began offering FP services through their Ministries of Health with Save the Children support earlier than the countries providing standard care. The DRC, Somalia, and Pakistan FP programs were launched in 2012, Yemen in 2013, and Rwanda and Syria in 2016. Thus, it is likely that newer country programs were still focused on overall start-up activities, whereas the more established programs were at a juncture where they could expand their work to focus on more specific areas of quality service delivery, including IPPFP. Similarly, all of the countries with higher IPPFP intensity interventions are in protracted crises, meaning that the population faces a higher risk of death, illness, and reduced livelihoods as a result of conflict, in the case of DRC and Somalia, and natural disaster, in the case of Pakistan. Meanwhile, Syria and Yemen are in more active, acute phases of conflict. This led to more notable restrictions; for instance, Ministry of Health staff from Somalia, DRC, Pakistan, and Rwanda were able to travel outside of their countries for specialized training on IPPFP, whereas staff from Syria and Yemen could not be due to visa and travel restrictions. Further, the acute crisis caused supply chain challenges for implant commodities in Syria and Yemen, which were not experienced in more protracted settings. Implants are not a part of the interagency reproductive health kits, which are relied on heavily for procurement of contraceptive supplies in acute emergencies, such as Yemen and Syria (27). Although implants can be procured external to the kits, it takes a great deal of time to get supplies into these two countries. The Rwanda program, although not an acute emergency, operates from a single health facility in a refugee camp, whereas the other five programs are in non-camp settings.

Although several countries have had ongoing success with PPIUD uptake, the practice still remains underutilized overall in humanitarian contexts. However, this study highlights that with sustained, multipronged approaches, demand for IPPFP can be generated and met. The integration of rights-based IPPFP services into existing reproductive health services allows for postpartum women in humanitarian settings to space their pregnancies in situations where it is often difficult to return to a health facility for FP in the future. This comparison study shows that by incorporating IPPFP training on counseling and service delivery for providers within maternity and ANC services, women, girls, and families will more readily choose to adopt an IPPFP method. IPPFP services are not overly complicated when quality FP services already exist within health facilities. These overall findings show that lessons learned from IPPFP development sector can be successfully applied to emergencies, increasing the opportunity to reduce maternal mortality in geographic locations where it is most concentrated.

## Data Availability Statement

The data analyzed in this study is subject to the following licenses/restrictions: The dataset can be made public for the publication; an internal approval process will be required. Requests to access these datasets should be directed to Meghan C. Gallagher, mgallagher@savechildren.org.

## Author Contributions

MG and CM conceived of the presented idea. RD and MG conducted the literature review. MG performed the computations. AF, AS, and BS provided programmatic inputs. MG and CM drafted the manuscript. All authors discussed the results and contributed to the final manuscript.

## Conflict of Interest

The authors declare that the research was conducted in the absence of any commercial or financial relationships that could be construed as a potential conflict of interest.

## References

[1] Cleland J Bernstein S Ezeh A Faundes A Glasier A Innis J. Family planning: the unfinished agenda. Lancet. (2006) 368:1810–27. 10.1016/S0140-6736(06)69480-4 17113431

[2] Erken A. Maternal deaths and humanitarian crises. Lancet. (2017) 389:1514. 10.1016/S0140-6736(17)30949-2 28422022

[3] Guttmacher Institute. In a State of Crisis: Meeting the Sexual and Reproductive Health Needs of Women in Humanitarian Situations. Guttmacher Policy Review. (2017). Available online at: https://www.guttmacher.org/gpr/2017/02/state-crisis-meeting-sexual-and-reproductive-health-needs-women-humanitarian-situations (accessed February 28, 2021).

[4] Casey S Chynoweth S Cornier N Gallagher M Wheeler E. Progress and gaps in reproductive health services in three humanitarian settings: mixed-methods case studies. BMC Conflict Health. (2015) 9:S3. 10.1186/1752-1505-9-S1-S3 25798189 PMC4331815

[5] Pasha O Goudar SS Patel A Garces A Esamai F Chomba E. Postpartum contraceptive use and unmet need for family planning in five low-income countries. BMC Reproduc Health. (2015) 12 (Suppl. 2):S11. 10.1186/1742-4755-12-S2-S11 26063346 PMC4464604

[6] Ho L Wheeler E. Using program data to improve access to family planning and enhance the method mix in conflict-affected areas of the democratic Republic of the Congo. Global Health Sci Practice. (2018) 6:161–77. 10.9745/GHSP-D-17-00365 29602870 PMC5878069

[7] High Impact Practices in Family Planning (HIPs). Immediate Postpartum Family Planning: A Key Component of Childbirth Care. Washington, DC: USAID (2017). Available online at: https://www.fphighimpactpractices.org/briefs/immediate-postpartum-family-planning/ (accessed February 28, 2021).

[8] Tran NT Seuc A Tshikaya B Mutuale M Landoulsi S Kini B. Effectiveness of post-partum family planning interventions on contraceptive use and method mix at 1 year after childbirth in Kinshasa, DR Congo (Yam Daabo): a single-blind, cluster-randomised controlled trial. Lancet Global Health. (2020) 8:e399–410. 10.1016/S2214-109X(19)30546-7 31958404 PMC7708388

[9] Stover J Ross J. How increased contraceptive use has reduced maternal mortality. Maternal Child Health J. (2010) 14:687–95. 10.1007/s10995-009-0505-y 19644742

[10] WHO. Family planning/Contraception. (2018). Available online at: https://www.who.int/news-room/fact-sheets/detail/family-planning-contraception (accessed February 28, 2021).

[11] The American College of Obstetricians and Gynecologists. Postpartum Birth Control. (2018). Available online at: https://www.acog.org/womens-health/faqs/postpartum-birth-control (accessed February 28, 2021).

[12] The American College of Obstetricians and Gynecologists. Immediate Postpartum Long-Acting Reversible Contraception. (2016). Available online at: https://www.acog.org/clinical/clinical-guidance/committee-opinion/articles/2016/08/immediate-postpartum-long-acting-reversible-contraception (accessed on February 28, 2021).

[13] Mayo Clinic. Family Planning: Get the Facts About Pregnancy Spacing. (2017). Available online at: https://www.mayoclinic.org/healthy-lifestyle/getting-pregnant/in-depth/family-planning/art-20044072 (accessed on February 28, 2021).

[14] Casey SE McNab SE Tanton C Odong J Testa AC Lee-Jones L. Availability of long-acting and permanent family-planning methods leads to increase in use in conflict-affected northern Uganda: evidence from cross-sectional baseline and endline cluster surveys. Glob Public Health. (2013) 8:284–97. 10.1080/17441692.2012.758302 23305269 PMC3613974

[15] MEASURE Evaluation. Healthy Timing and Spacing of Pregnancy. Available online at: https://www.measureevaluation.org/prh/rh_indicators/family-planning/htsp/healthy-timing-and-spacing-of-pregnancies.html (accessed on February 28, 2021).

[16] World Health Organization. Medical Eligibility Criteria for Contraceptive Use, 5th ed. Geneva: World Health Organization (2015). Available online at: https://apps.who.int/iris/rest/bitstreams/1243459/retrieve (accessed February 28, 2021).

[17] The American College of Obstetricians and Gynecologists. Long-Acting Reversible Contraception: Intrauterine Device and Implant. (2018). Available online at: https://www.acog.org/womens-health/faqs/long-acting-reversible-contraception-iud-and-implant (accessed February 28, 2021).

[18] Washington CI Jamshidi R Thung SF Nayeri UA Caughey AB Werner EF. Timing of postpartum intrauterine device placement: a cost-effectiveness analysis. Fertil Steril. (2015) 103:131–7. 10.1016/j.fertnstert.2014.09.032 25439838

[19] Gariepy A Duffy J Xu X. Cost-effectiveness of immediate compared with delayed postpartum etonogestrel implant insertion. Obstet Gynecol. (2015) 126:47–55. 10.1097/AOG.0000000000000907 26241255 PMC4526123

[20] World Health Organization. HTSP 101: Everything You Want to Know About Healthy Timing and Spacing of Pregnancy. Available online at: https://www.who.int/pmnch/topics/maternal/htsp101.pdf (accessed February 28, 2021).

[21] McGinn T Austin J Anfinson K Amsalu R Casey SE Fadulalmula SI. Family planning in conflict: results of cross-sectional baseline surveys in three African countries. Conflict Health. (2011) 5:11. 10.1186/1752-1505-5-11 21752241 PMC3162885

[22] World Health Organization. Improving Family Planning Service Delivery in Humanitarian Crises. (2018). Available online at: https://www.who.int/reproductivehealth/publications/family_planning/family-planning-humanitarian-crises/en/ (accessed February 28, 2021).

[23] Casey SE Tshipamba M. Contraceptive availability leads to increase in use in conflict-affected Democratic Republic of the Congo: evidence from cross-sectional cluster surveys, facility assessments and service statistics. Confl Health. (2017) 11:2. 10.1186/s13031-017-0104-2 28286546 PMC5341463

[24] United Nations. Contraceptive Use by Method 2019: Data Booklet. New York, NY: United Nations (2019). 10.18356/1bd58a10-en

[25] Mowafi H Nowak K Hein K. Facing the challenges in human resources for humanitarian health. Prehosp Disast Med. (2007) 22:351. 10.1017/S1049023X00005057 18087902

[26] Sarker M Matin M Mehjabeen S Tamim MA Sharkey AB Kim M. Effective maternal, newborn and child health programming among Rohingya refugees in Cox's Bazar, Bangladesh: Implementation challenges and potential solutions. PLoS ONE. (2020) 15:e0230732. 10.1371/journal.pone.0230732 32214359 PMC7098630

[27] Foster AM Evans DP Garcia M Knaster S Krause S McGinn T. The 2018 Inter-agency field manual on reproductive health in humanitarian settings: revising the global standards. Reprod Health Matters. (2017) 25:18–24. 10.1080/09688080.2017.1403277 29231788

